# ALKBH5 facilitates acute myeloid leukemia development and immune escape via PD-L1 regulation

**DOI:** 10.3389/fonc.2026.1781803

**Published:** 2026-02-26

**Authors:** Xin Ma, Cong Zhao, Ying Xi, Le Fu, Yuemei Feng, Yan Wang, Xiangmei Ye, Haiyan Gao

**Affiliations:** 1 Department of Clinical Laboratory, The Sixth Affiliated Hospital of Harbin Medical University, Harbin, China; 2 Department of Microbiology, Harbin Medical University, Harbin, China; 3 Department of Laboratory Diagnostics, The First Affiliated Hospital of Harbin Medical University, Harbin, China

**Keywords:** acute myeloid leukemia, ALKBH5, N6-methyladenosine (m6A), PD-L1, tumor immune microenvironment

## Abstract

**Background:**

Acute myeloid leukemia (AML) is a highly heterogeneous malignant hematological tumor, and its occurrence and development are closely related to immune evasion. Programmed death ligand 1 (PD-L1) is a key molecule mediating tumor immune escape, but its regulatory mechanism in AML has not been fully elucidated. The m6A demethylase ALKBH5 is highly expressed in various cancers, including AML, and promotes disease progression, but its specific role in modulating the PD-L1 axis and the immune microenvironment in AML remains unclear.

**Methods:**

We analyzed the expression and prognostic significance of ALKBH5 in AML by integrating BloodSpot, GEO, and TCGA databases, and performed functional enrichment and immune infiltration analyses. Bone marrow samples from 77 newly diagnosed AML patients and 13 healthy controls were collected for RT-qPCR validation, and their correlations with clinical features and PD-L1 expression were evaluated. ALKBH5 stable knockdown AML cell lines (THP-1 and MOLM-13) were constructed to assess cell proliferation, migration, and PD-L1 expression. T cell anti-tumor effects were evaluated using an AML cell and activated PBMC co-culture system.

**Results:**

Bioinformatics analysis shows that ALKBH5 is significantly overexpressed in AML and is associated with poor prognosis and enrichment of immune related signaling pathways. Clinical sample testing further confirms that the level of ALKBH5 in AML patients is elevated, which is positively correlated with an increase in the proportion of primitive cells and PD-L1 expression. *In vitro* experiments have shown that ALKBH5 knockdown significantly inhibits AML cell proliferation and migration, while reducing PD-L1 protein levels without affecting its mRNA expression, suggesting that it regulates PD-L1 through post transcriptional mechanisms. Co-culture experiments showed that ALKBH5 knockdown enhanced the proportion of CD8^+^ T cells and secretion of IFN-γ/TNF-α, and reduced the survival rate of AML cells.

**Conclusions:**

ALKBH5 may promote AML progression and immune escape through the upregulation of PD-L1 and modulation of T-cell function, which provides a theoretical basis for the development and screening of novel immunotherapeutic strategies for AML.

## 1 Introduction

Acute myeloid leukemia (AML) is a highly heterogeneous malignant hematologic disease originating from hematopoietic stem and progenitor cells, characterized by abnormal proliferation of myeloid cells, differentiation blockade, and impaired normal hematopoietic function of the bone marrow (1–3). Despite significant advances in targeted therapies and immunotherapies in recent years, the overall survival of AML patients remains suboptimal, particularly among elderly individuals and those with relapsed or refractory disease, who exhibit extremely poor prognosis (4, 5). Therefore, it is urgent to develop new strategies to prevent AML progression and improve patient prognosis. Research has shown that immune escape is a key mechanism for AML progression and treatment resistance (6, 7). Leukemia cells achieve sustained growth and evade immune clearance by shaping an immunosuppressive bone marrow immune microenvironment, including T cell depletion, myeloid derived suppressor cell expansion, and impaired antigen presentation function, thereby limiting the efficacy of traditional therapies and immunotherapy (8, 9). Therefore, in-depth analysis of the pathogenesis of AML, especially the key pathways of immune escape, is of great significance for the development of new treatment strategies.

Programmed Death-Ligand 1 (PD-L1) is an important molecule for tumor cells to evade immune surveillance. It binds to PD-1 on the surface of T cells, inhibits T cell activation, proliferation, and cytokine secretion, thereby forming an immunosuppressive microenvironment (10, 11). In AML, PD-L1 expression is regulated by multiple mechanisms, including TP53 mutation–induced MYC upregulation and miR-34a downregulation (12), inflammatory cytokine signaling such as IFN-γ and TNF-α through STAT1/STAT3 and MEK pathways (13), and epigenetic modulation by microRNAs like miR-200c (14). These mechanisms not only promote immune evasion but also confer proliferative advantages to PD-L1+ AML blasts via enhanced glycolysis and cell cycle progression through CD274/JNK/Cyclin D2 signaling (15). Moreover, PD-L1 upregulation has been linked to resistance against hypomethylating agents (HMAs) (16), further contributing to poor prognosis in AML patients. Additional studies also show associations between FLT3-ITD/NPM1 mutations and PD-L1 upregulation (17). However, the regulatory mechanisms of PD-L1 expression in AML remain incompletely characterized, particularly post-transcriptional epigenetic regulation.

N^6^-methyladenosine (m6A) is the most abundant internal chemical modification in eukaryotic mRNA and can dynamically regulate RNA stability, splicing, translation, and degradation, playing critical roles in various physiological and pathological processes (18, 19). Among them, the demethylase ALKBH5 acts as a critical regulatory factor and influences immune-related pathways in various cancers. For example, ALKBH5 stabilizes PD-L1 mRNA through demethylation to suppress T cell function (20); promotes the recruitment and polarization of immunosuppressive macrophages by upregulating inflammatory cytokines (21, 22); and enhances the infiltration of regulatory T cells and myeloid-derived suppressor cells by altering lactate metabolism (23). There is evidence that ALKBH5 is highly expressed in AML and closely associated with poor prognosis; by enhancing the mRNA stability of key genes such as TACC3 and AXL, it promotes leukemic cell proliferation and impedes differentiation (24, 25). However, whether ALKBH5 participates in PD-L1 regulation and immune microenvironment remodeling in AML remains unclear.

This study aims to elucidate the m6A-dependent molecular mechanism by which ALKBH5 regulates PD-L1 expression and to clarify its critical role in mediating immune escape in AML. These findings not only expand the understanding of the function of m6A modification within the leukemic immune microenvironment but also provide a theoretical basis for the development of ALKBH5-targeted therapies and for enhancing the efficacy of existing immune checkpoint inhibitors.

## 2 Materials and methods

### 2.1 Bioinformatic analysis

All bioinformatic analyses in this study were performed using the R software environment (version 4.4.1). The BloodSpot database (https://www.bloodspot.eu/) was utilized to integrate the GSE13159 and GSE42519 datasets and to compare ALKBH5 expression levels between AML patients and normal controls. RNA-Seq expression data and corresponding clinical information for the TCGA-LAML cohort were retrieved from The Cancer Genome Atlas (TCGA) database to conduct survival analysis of ALKBH5. The AML dataset GSE13159 (GPL570 platform) was obtained from the GEO database for differential expression analysis, functional enrichment, and immune infiltration analysis. Differentially expressed genes (DEGs) between high- and low-ALKBH5 expression groups were identified using the limma R package, with the selection criteria defined as |log_2_FC| > 0.3 and adjusted *P* value < 0.05. Gene Ontology (GO) and Kyoto Encyclopedia of Genes and Genomes (KEGG) enrichment analyses of the identified DEGs were conducted using the clusterProfiler package. The CIBERSORT algorithm was applied to the normalized expression matrix of the GSE13159 dataset to estimate the proportions of 22 immune cell subtypes. Spearman correlation analysis was subsequently performed to assess the associations between ALKBH5 expression, immune cell infiltration levels, and the expression of key immune checkpoint molecules.

### 2.2 Patients and clinical samples

This study enrolled 77 newly diagnosed AML patients and 13 non-hematologic disease controls who were admitted to the First Affiliated Hospital of Harbin Medical University between January 2020 and June 2024 (**Supplementary Table 1**). All study protocols were reviewed and approved by the Ethics Committee of Harbin Medical University (approval number: hrbmuecdc20251201), and written informed consent was obtained from all participants. Bone marrow samples were collected from all subjects, and bone marrow mononuclear cells (BMNCs) were isolated using Ficoll (Solarbio, Beijing, China) density gradient centrifugation for subsequent total RNA extraction. Clinical data collected included age, sex, French-American-British (FAB) classification, chromosomal karyotype, proportions of bone marrow and peripheral blood blasts, white blood cell count, and lymphocyte percentages determined by flow cytometry.

### 2.3 Cell culture

The human acute myeloid leukemia cell lines THP-1 and MOLM-13 were generously provided by Tongji Hospital, Huazhong University of Science and Technology. The human bone marrow stromal cell line HS-5 was purchased from Procell Life Science & Technology Co., Ltd. (Wuhan, China). THP-1 and MOLM-13 cells were cultured in RPMI-1640 medium (Gibco, Shanghai, China) supplemented with 10% heat-inactivated fetal bovine serum (FBS, Abbkine, USA) and 1% penicillin-streptomycin (Beyotime, Shanghai, China). HS-5 cells were maintained in DMEM medium (Gibco, Shanghai, China) with the same supplements. All cells were incubated at 37 °C in a humidified incubator containing 5% CO_2_.

### 2.4 Lentiviral transduction and stable cell line generation

To establish ALKBH5 stable knockdown cell models, three specific short hairpin RNAs (shRNAs) and one negative control shRNA (sh-NC) were synthesized and packaged into lentiviruses by General Biol Co., Ltd. (Hefei, China). Polybrene (General Biol, Hefei, China) was added during transduction to enhance infection efficiency. Seventy-two hours post-transduction, cells were selected in medium containing 4 μg/mL puromycin (Beyotime, Shanghai, China) until the control group cells were completely eliminated. Knockdown efficiency was confirmed by RT-qPCR and Western blot analyses. The sequences of shRNAs used in this study are listed in **Supplementary Table 2**.

### 2.5 Real-time quantitative PCR

Total RNA was extracted from cells using TRIzol reagent (Takara, Beijing, China). RNA concentration and purity were determined using a NanoDrop 2000C spectrophotometer (Thermo Fisher Scientific, MA, USA). One microgram of total RNA was treated with the PrimeScript™ RT reagent Kit with gDNA Eraser (Takara, Beijing, China) to remove genomic DNA and reverse transcribed into cDNA. Real-time quantitative PCR was performed using the SYBR^®^ Green Kit (Roche, Basel, Switzerland) on an SLAN-96P real-time PCR system (Hongshi, Shanghai, China). The thermal cycling conditions were as follows: 95 °C for 30 s, followed by 40 cycles of 95 °C for 5 s and 60 °C for 30 s. β-actin was used as the internal reference gene, and the relative expression of target genes was calculated using the 2^-ΔΔCt^ method. The primer sequences used in this study are listed in **Supplementary Table 3**.

### 2.6 Western blot

Total protein was extracted from cells using RIPA lysis buffer supplemented with protease inhibitors (Beyotime, Shanghai, China), and protein concentration was determined using a BCA Protein Assay Kit (Beyotime, Shanghai, China). Thirty micrograms of protein were separated on 10% SDS-PAGE gels and then transferred onto nitrocellulose membranes using the wet transfer method (LABSELECT, Shanghai, China). The membranes were blocked with 5% skimmed milk (Biosharp, Beijing, China) for 1 hour and incubated overnight at 4 °C with the respective primary antibodies: rabbit polyclonal anti-ALKBH5 (1:5000, Origene, Wuxi, China), rabbit monoclonal anti-PD-L1 (1:2000, Proteintech, Wuhan, China), and mouse monoclonal anti-GAPDH (1:2000, Origene, Wuxi, China). The following day, membranes were incubated with HRP-conjugated secondary antibody goat anti-mouse IgG (1:10000, Abbkine, Wuhan, China) at room temperature for 1 hour. Signals were detected using ECL chemiluminescent substrate (Abbkine, Wuhan, China) and captured with the Tanon automated chemiluminescence imaging system (Tanon Image System, Shanghai, China). Band intensities were quantified using ImageJ software (NIH, Bethesda, MD, USA), and the signal of each target protein was normalized to the corresponding internal control.

### 2.7 Cell proliferation assay

Cell proliferation was evaluated using the Cell Counting Kit-8 (CCK-8, Abbkine, Wuhan, China). Cells were seeded into 96-well plates at a density of 1×10^4^ cells per well. At 24, 48, and 72 hours post-seeding, 10 μL of CCK-8 reagent was added to each well. After incubation for 1.5 hours, absorbance was measured at 450 nm. For each time point and condition, six replicate wells were set up, and the entire experiment was independently repeated three times.

### 2.8 Cell migration assay

Cell migration was assessed using Transwell chambers with 8.0 μm pores (LABSELECT, Shanghai, China). A total of 1×10^5^ cells, starved in serum-free medium for 24 hours, were resuspended and added to the upper chamber. The lower chamber was filled with complete medium containing 10% FBS as a chemoattractant. After 48 hours of incubation, cells that had migrated to the lower chamber were collected and quantified using the CCK-8 assay.

### 2.9 Peripheral blood mononuclear cell isolation and *in vitro* T cell cytotoxicity assay

Peripheral blood was collected from healthy donors, and peripheral blood mononuclear cells (PBMCs) were isolated using Ficoll density gradient centrifugation (Solarbio, Beijing, China). PBMCs were resuspended in complete medium and activated with ImmunoCult™ Human CD3/CD28 T Cell Activator (STEMCELL Technologies, Shanghai, China) and recombinant human IL-2 (final concentration 20 ng/mL, Abbkine, Wuhan, China) for 3–5 days for subsequent experiments.

Activated PBMCs were co-cultured with MOLM-13 cells at an effector-to-target (E:T) ratio of 4:1 in 24-well plates, with target cells (MOLM-13) seeded at 1×10^5^ cells per well and effector cells (PBMCs) at 4×10^5^ cells per well. Cells and supernatants were collected at 24 and 48 hours. For flow cytometry analysis, cells were surface-stained with PE anti-CD3, FITC anti-CD4, and APC anti-CD8 antibodies (Elabscience, Wuhan, China), and analyzed using a FACSCanto™ II flow cytometer (BD Biosciences, Franklin Lakes, NJ, USA). Concentrations of IFN-γ and TNF-α in the supernatants were measured using ELISA kits (Elabscience, Wuhan, China), and the survival rate of AML cells post co-culture was assessed using the CCK-8 assay.

### 2.10 Statistical analysis

All statistical analyses were performed using GraphPad Prism 9.0 and R software (v4.4.1). Quantitative data conforming to a normal distribution are presented as mean ± standard deviation (SD), and comparisons between two groups were performed using Student’s t-test, while comparisons among multiple groups were conducted using one-way analysis of variance (ANOVA). Non-normally distributed data are presented as median and compared using the Mann-Whitney U test. Categorical data were analyzed using the chi-square test. Correlation analyses were conducted using Pearson or Spearman correlation based on data distribution. Survival analyses were performed using the Kaplan-Meier method and compared with the log-rank test. *P* < 0.05 was considered statistically significant.

## 3 Results

### 3.1 Bioinformatic analysis reveals high ALKBH5 expression, poor prognosis, and immune regulatory potential in AML

**Figure 1:**
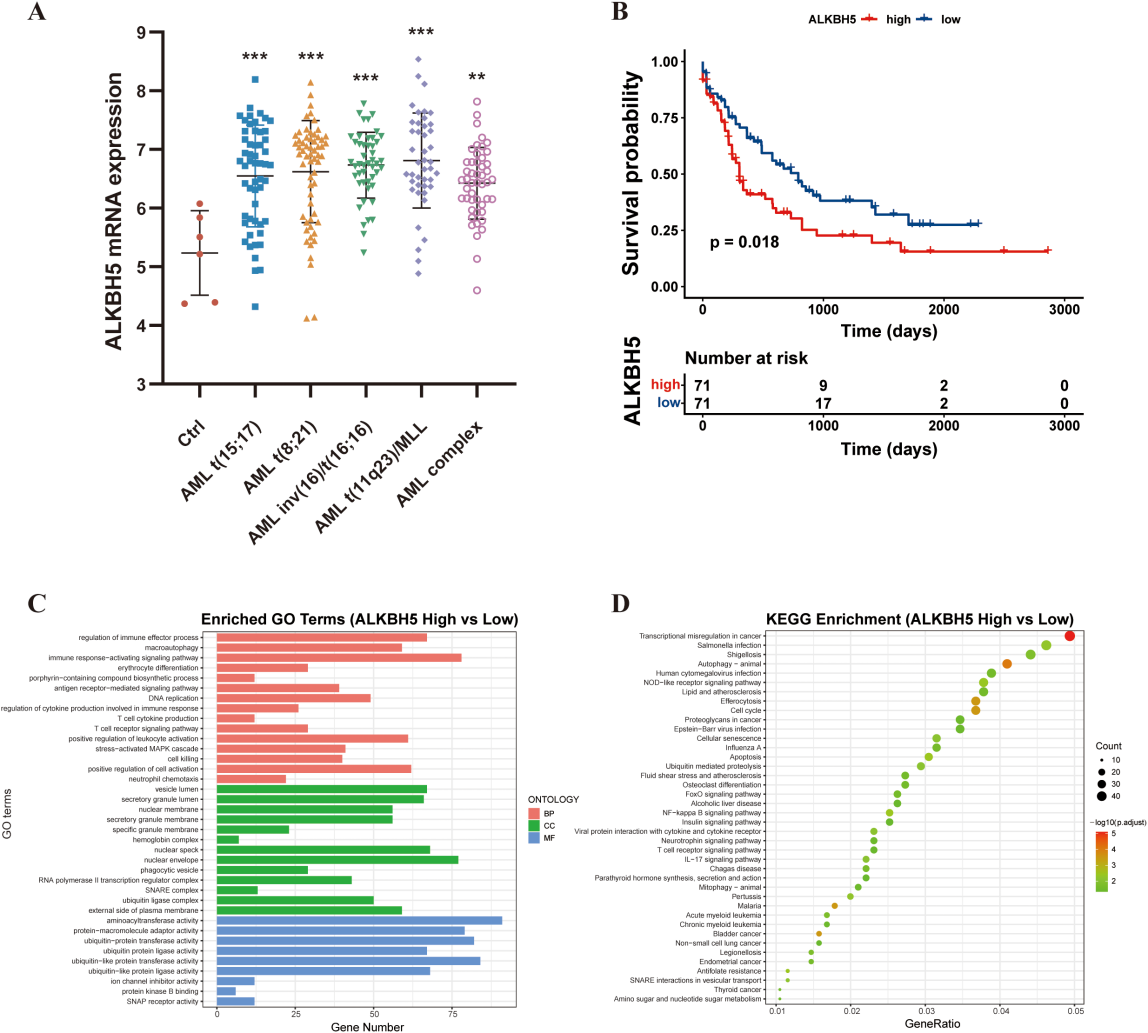
Expression pattern, prognostic significance, and functional enrichment of ALKBH5 in acute myeloid leukemia (AML). **(A)** ALKBH5 expression levels in primary AML patients carrying distinct chromosomal translocations compared with bone marrow hematopoietic stem cells (HSCs, Ctrl) from healthy donors, based on the GSE13159 and GSE42519 datasets. **(B)** Kaplan–Meier overall survival analysis of AML patients in the TCGA-LAML cohort stratified into ALKBH5 high- and low-expression groups according to the median ALKBH5 expression. **(C)** Gene Ontology (GO) enrichment analysis of differentially expressed genes between ALKBH5 high- and low-expression groups. **(D)** KEGG pathway enrichment bubble plot of differentially expressed genes between ALKBH5 high- and low-expression groups. ns, not significant; *P < 0.05; **P < 0.01; ***P < 0.001.

To systematically investigate the role of ALKBH5 in AML, we first integrated GSE13159 and GSE42519 datasets via the BloodSpot database, which revealed that ALKBH5 expression was markedly elevated in primary AML patients bearing various chromosomal translocations compared to normal hematopoietic stem cells (**Figure 1A**). Subsequently, we performed bioinformatic analyses. Survival analysis of the TCGA-LAML cohort demonstrated that patients were stratified into high- and low-ALKBH5 expression groups based on the median ALKBH5 expression level, and those with high ALKBH5 expression exhibited significantly shorter overall survival compared with patients in the low-expression group. (*P* < 0.01, **Figure 1B**).

To explore the biological functions associated with high ALKBH5 expression, differential gene expression analysis between ALKBH5 high- and low-expression groups was conducted, followed by Gene Ontology (GO) and Kyoto Encyclopedia of Genes and Genomes (KEGG) enrichment analyses. The results showed that differentially expressed genes (DEGs) were significantly enriched in immune-related processes, including positive regulation of leukocyte activation, T cell cytokine production, and regulation of cytokine production involved in immune response (**Figure 1C**). Pathway analysis further revealed enrichment in key immune signaling pathways such as NF-κB signaling, T cell receptor signaling, and IL-17 signaling (**Figure 1D**), suggesting a potential role for ALKBH5 in modulating immune regulation in AML.

**Figure 2:**
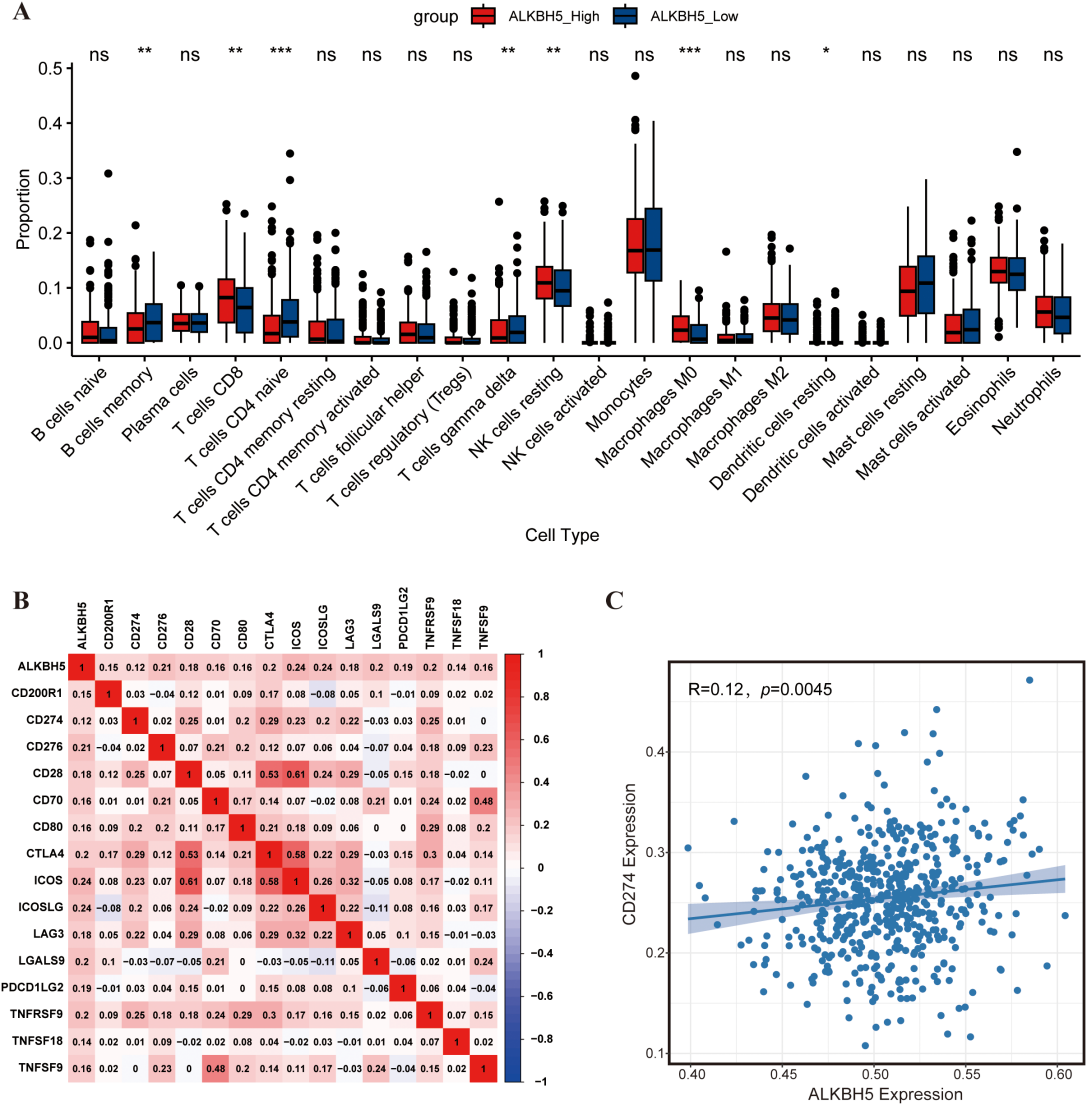
Immune infiltration and immunological correlation analyses in the GSE13159 dataset. **(A)** Box plots showing the relative proportions of immune cell subtypes estimated by the CIBERSORT algorithm in AML samples with high versus low ALKBH5 expression. Samples were stratified into high- and low-ALKBH5 expression groups based on the median ALKBH5 expression level. Differences between groups were assessed using the Wilcoxon rank-sum test. **(B)** Pearson correlation heatmap illustrating the associations between ALKBH5 expression and multiple immune checkpoint molecules in AML samples. **(C)** Pearson correlation analysis between ALKBH5 and CD274 (PD-L1) expression in AML patients. ns, not significant; *P < 0.05; **P < 0.01; ***P < 0.001.

Subsequently, we applied the CIBERSORT algorithm to assess the relationship between ALKBH5 expression and the tumor immune microenvironment. High ALKBH5 expression was significantly positively correlated with the infiltration levels of CD8^+^ T cells, resting NK cells, and M0 macrophages (**Figure 2A**, **Supplementary Figure 1**). Notably, ALKBH5 expression was strongly positively associated with several key immune checkpoint molecules, including PD-L1, CTLA-4, and LAG3 (**Figures 2B, C**). In summary, these bioinformatics results suggest that ALKBH5 is highly expressed in AML and may promote disease progression by regulating the immune microenvironment. Based on this, we hypothesize that ALKBH5 may mediate immune escape in AML by up-regulating PD-L1. To verify this hypothesis, we further conducted *in vitro* functional and mechanistic studies.

### 3.2 Clinical validation of ALKBH5 expression and its association with tumor burden and PD-L1 in AML

**Figure 3:**
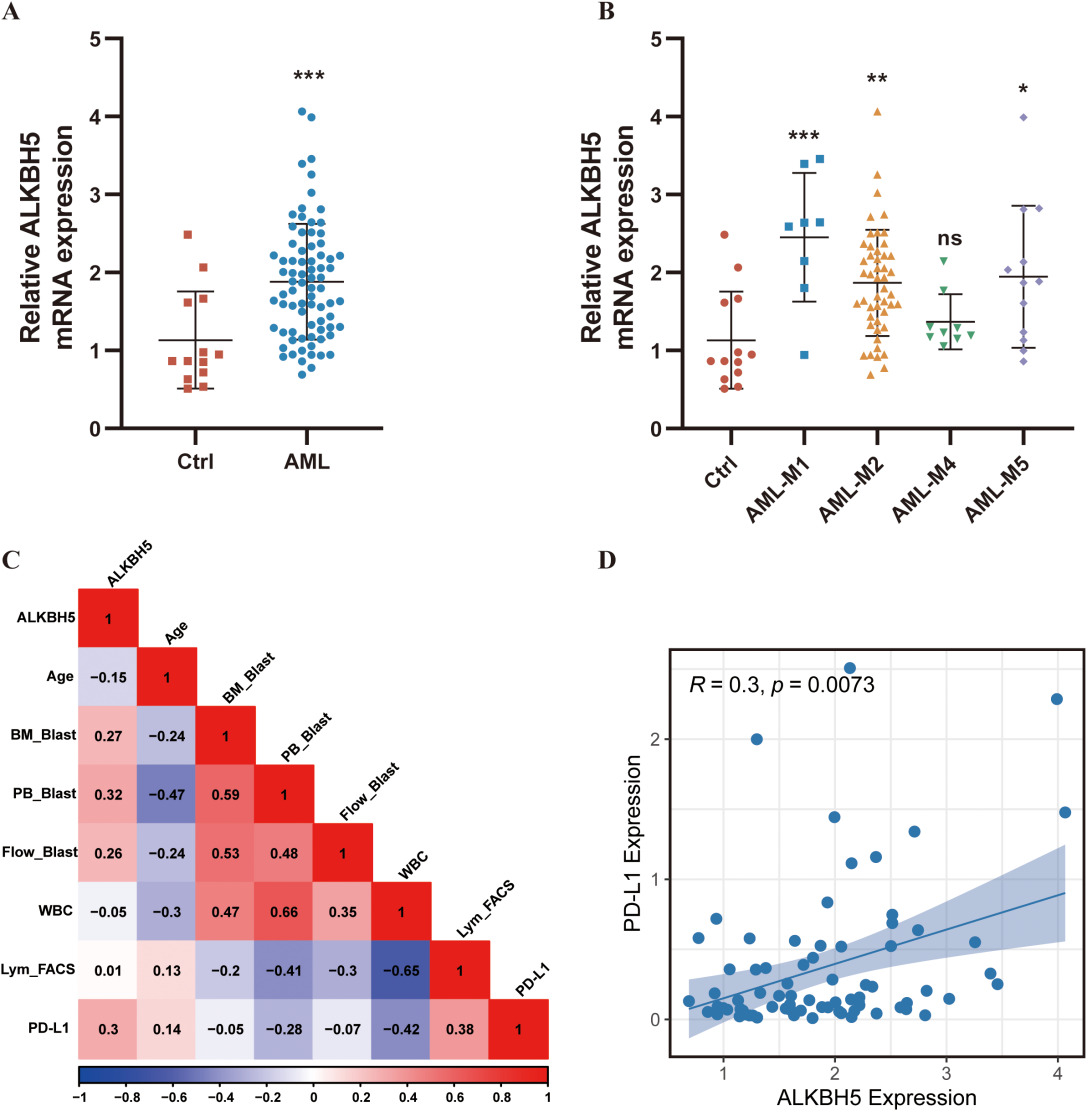
Analysis of clinical samples. **(A)** RT-qPCR analysis of ALKBH5 expression in bone marrow cells from healthy donors (n = 13) and AML patients (n = 77) **(B)** RT-qPCR analysis of ALKBH5 expression in bone marrow cells from healthy donors (n = 13) and AML patients stratified by different FAB subtypes (n = 77) **(C)** Spearman correlation heatmap showing the associations between ALKBH5 expression levels and clinical characteristics in AML patients (n = 77). Age: patient age (years); BM_Blast: percentage of blasts in bone marrow (%); PB_Blast: percentage of blasts in peripheral blood (%); Flow_Blast: percentage of blasts in bone marrow detected by flow cytometry (%); WBC: white blood cell count (×10^9^/L); Lym_FACS: percentage of lymphocytes in bone marrow measured by flow cytometry (%); **(D)** Spearman correlation analysis between ALKBH5 and PD-L1 expression in AML patients (n = 77). ns, not significant; *P < 0.05; **P < 0.01; ***P < 0.001.

To validate the aforementioned bioinformatic findings, bone marrow samples from 77 newly diagnosed AML patients and 13 healthy controls were collected for experimental verification. RT-qPCR results confirmed that ALKBH5 mRNA levels were significantly elevated in AML patient samples compared with healthy controls (*P* < 0.001, **Figure 3A**), with the highest expression observed in the M1 subtype and the lowest in M4 (**Figure 3B**). AML patients were stratified into high- and low-ALKBH5 expression groups based on the median expression level, and their clinical characteristics were analyzed. Patients in the ALKBH5 high-expression group exhibited a significantly higher proportion of peripheral blood blasts (58.00% vs. 33.50%, *P* = 0.02) and a trend toward higher bone marrow blast percentage (51.00% vs. 44.00%, *P* = 0.05), indicating a close association between ALKBH5 expression and tumor burden in AML (**Table 1**, **Figure 3C**, **Supplementary Figure 2**). Notably, the proportion of lymphocytes in the bone marrow (assessed by flow cytometry) was slightly higher in the ALKBH5 high-expression group than in the low-expression group, though the difference was not statistically significant (6.50% vs. 5.39%, *P* = 0.72), which may be attributable to limited sample size. More importantly, clinical sample data successfully validated the bioinformatic prediction: ALKBH5 expression was significantly positively correlated with PD-L1 mRNA levels (r = 0.30, *P* = 0.0073, **Figure 3D**). Additionally, PD-L1 expression levels were significantly higher in the ALKBH5 high-expression group compared with the low-expression group (*P* = 0.01). These clinical findings directly link elevated ALKBH5 expression to the key immune evasion molecule PD-L1 in AML, providing a clear direction for subsequent mechanistic investigations.

**Table 1:** Association of ALKBH5 mRNA expression with clinical features in AML patients.

Characteristics	ALKBH5 high n=39	ALKBH5 low n=38	*P* value
Age (years)	54.00 (43.50, 66.00)	62.00 (48.25, 68.00)	0.43
Gender, N (%)			0.56
Female	19 (48.72%)	15 (39.47%)	
Male	20 (51.28%)	23 (60.53%)	
RBC (×10¹²/L), Mean (SD)	2.63 (0.83)	2.42 (0.74)	0.25
Hb (g/L), Mean (SD)	84.69 (27.87)	81.17 (23.82)	0.55
WBC (×10^9^/L) Median (Q1–Q3)	19.32 (3.43, 47.53)	18.69 (6.76, 47.61)	0.83
PLT (×10^9^/L) Median (Q1–Q3)	43.21 (17.72, 76.18)	45.57 (18.45, 68.00)	0.77
BM blasts (%) Median (Q1–Q3)	51.00 (31.75, 85.50)	44.00 (28.13, 63.13)	0.05
PB blasts (%) Median (Q1–Q3)	58.00 (16.50, 83.50)	33.50 (14.00, 54.75)	**0.02**
Flow blasts (%) Median (Q1–Q3)	66.76 (35.77, 83.58)	35.17 (18.71, 78.34)	0.06
Lym FACS (%) Median (Q1–Q3)	6.50 (3.49, 11.41)	5.39 (3.71, 9.31)	0.72
PD-L1 expression Median (Q1–Q3)	0.25 (0.10, 0.66)	0.09 (0.06, 0.24)	**0.01**
FAB classification, N (%)			**0.02**
M1	7 (17.95%)	1 (2.63%)	
M2	24 (61.54%)	24 (63.16%)	
M4	1 (2.56%)	8 (21.05%)	
M5	7 (17.95%)	5 (13.16%)	
Karyotype, N (%)			0.48
Abnormal	5 (12.82%)	8 (21.05%)	
Normal	12 (30.77%)	8 (21.05%)	
Unknown	22 (56.41%)	22 (57.89%)	

RBC (×10^12^/L): red blood cell count; Hb (g/L): hemoglobin concentration; WBC (×10^9^/L): white blood cell count; PLT (×10^9^/L): platelet count; BM blasts (%): percentage of blasts in bone marrow; PB blasts (%): percentage of blasts in peripheral blood; Flow blasts (%): percentage of blasts in bone marrow detected by flow cytometry; Lym FACS (%): percentage of lymphocytes in bone marrow measured by flow cytometry; FAB classification: French-American-British classification of AML subtypes (M1–M5); Karyotype: chromosomal analysis (Abnormal, Normal, Unknown). Values are presented as mean ± SD or median (Q1–Q3), as indicated.Bold values indicate statistical significance (*P* < 0.05).

### 3.3 *In vitro* functional assays confirm high ALKBH5 expression and its role in promoting malignant phenotypes in AML cells

**Figure 4:**
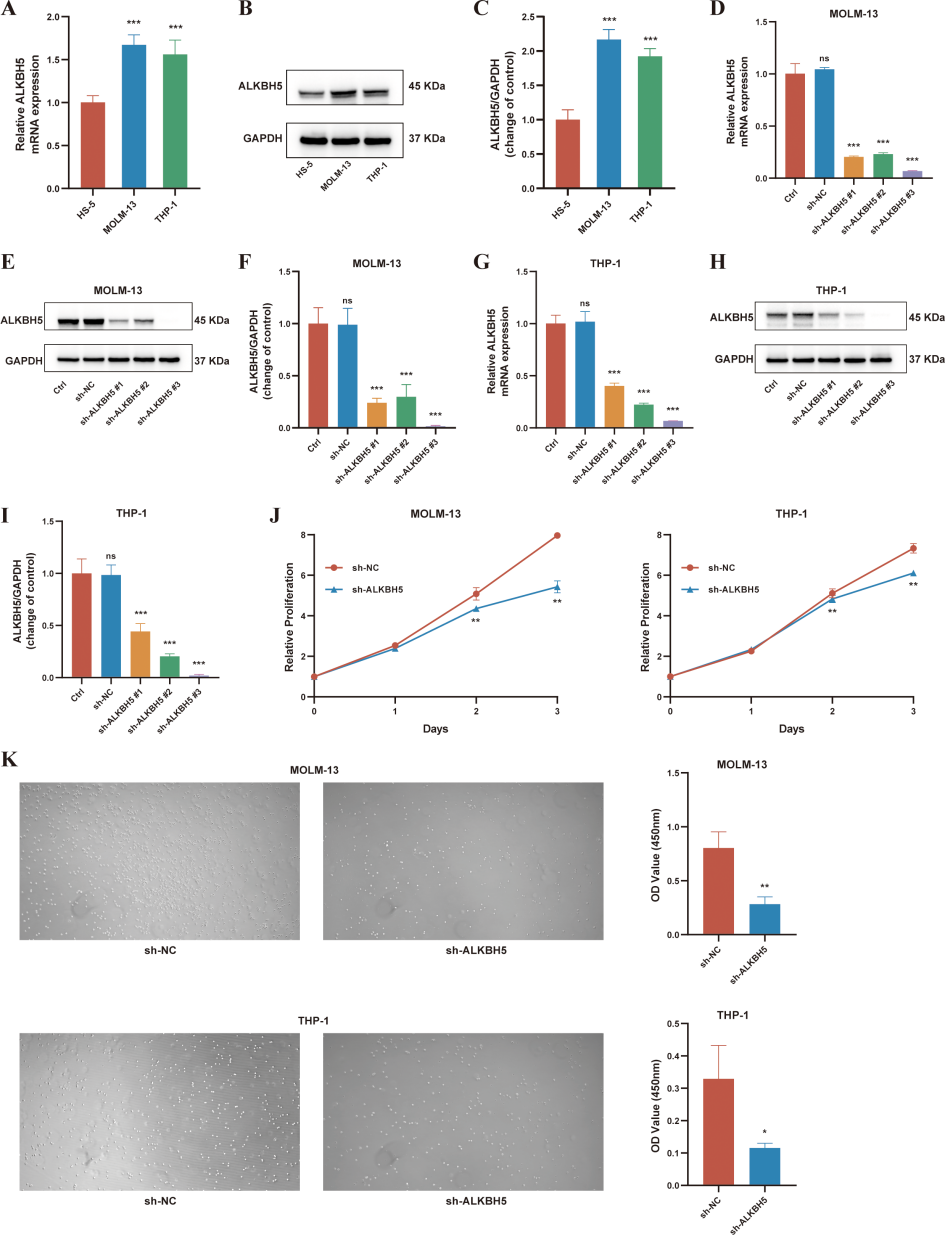
Knockdown of ALKBH5 suppresses proliferation and migration in AML cell lines. **(A–C)** Validation of ALKBH5 expression in normal bone marrow stromal cells (HS-5) and AML cell lines (MOLM-13, THP-1) by RT-qPCR **(A)** and Western blot **(B, C)**. **(D–F)** Verification of ALKBH5 knockdown efficiency in MOLM-13 cells transduced with sh-ALKBH5 or negative control shRNA (sh-NC) by RT-qPCR **(D)** and Western blot **(E, F)**. **(G–I)** Verification of ALKBH5 knockdown efficiency in THP-1 cells transduced with sh-ALKBH5 or sh-NC by RT-qPCR **(G)** and Western blot **(H, I)**. **(J)** CCK-8 assay evaluating the proliferative capacity of MOLM-13 and THP-1 cells with sh-ALKBH5 or sh-NC transduction at 24 h, 48 h, and 72 h. **(K)** Transwell migration assay evaluating the migratory capacity of MOLM-13 and THP-1 cells with sh-ALKBH5 or sh-NC transduction, quantified by the CCK-8 assay. ns, not significant; *P < 0.05; **P < 0.01; ***P < 0.001.

To validate the expression pattern and functional role of ALKBH5 at the cellular level, we first assessed ALKBH5 expression across multiple cell lines. Both RT-qPCR and Western blot analyses consistently demonstrated that ALKBH5 mRNA and protein levels were significantly upregulated in AML cell lines (THP-1, MOLM-13) compared with normal bone marrow stromal cells (HS-5) (**Figures 4A–C**), which is highly consistent with our clinical sample findings.

To investigate the biological function of ALKBH5, lentivirus-mediated shRNA was used to knock down ALKBH5 in THP-1 and MOLM-13 cells. Three distinct shRNA sequences (sh-ALKBH5#1, #2, #3) were constructed. Both RT-qPCR and Western blot confirmed that all three shRNAs effectively reduced ALKBH5 expression compared with the negative control (sh-NC), with sh-ALKBH5#3 exhibiting the most stable and pronounced knockdown across all cell lines (**Figures 4D–I**). Therefore, subsequent experiments employed the stable knockdown model generated using sh-ALKBH5#3. Functional assays revealed that ALKBH5 knockdown significantly inhibited AML cell proliferation (*P* < 0.01, **Figure 4J**) and migration (*P* < 0.001, **Figure 4K**), confirming the direct role of ALKBH5 in driving malignant progression in AML. These findings establish the role of ALKBH5 in driving the intrinsic malignant behavior of AML cells. Based on pathway enrichment analysis, we further speculate that ALKBH5 may also facilitate the immune evasion of AML cells, which could represent another critical mechanism contributing to poor patient prognosis.

### 3.4 ALKBH5 upregulates PD-L1 via a post-transcriptional mechanism and suppresses CD8^+^ T cell function

**Figure 5:**
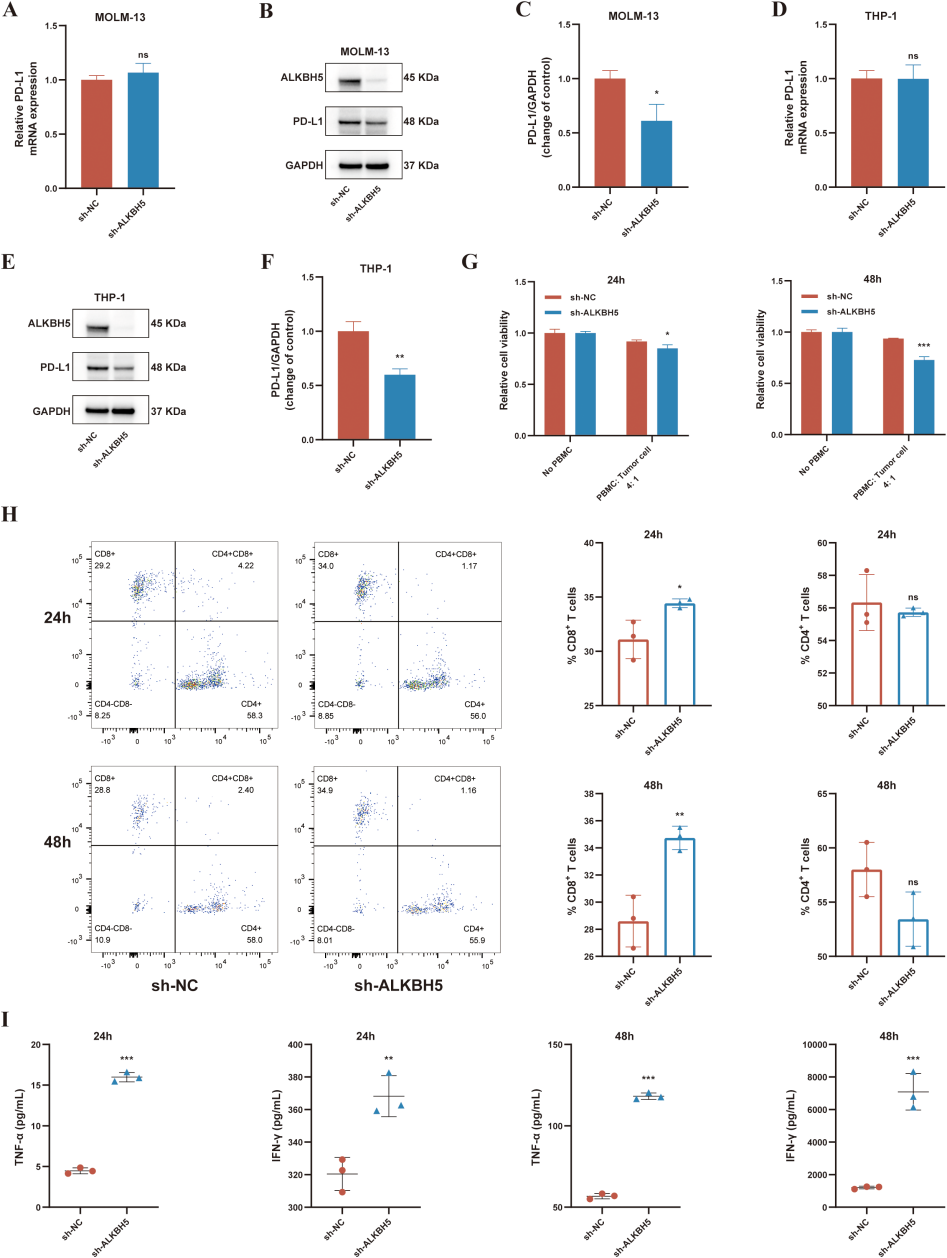
ALKBH5 knockdown regulates PD-L1 expression and enhances anti-tumor immune responses in AML cells. **(A–C)** PD-L1 expression in MOLM-13 cells after stable ALKBH5 knockdown measured by RT-qPCR **(A)** and Western blot **(B, C)**. **(D–F)** PD-L1 expression in THP-1 cells after ALKBH5 knockdown measured by RT-qPCR **(D)** and Western blot **(E, F)**. **(G)** CCK-8 assay evaluating the survival rate of sh-ALKBH5 or sh-NC transduced AML cells after 24 h and 48 h of co-culture with PBMCs. Viability was normalized to AML cells cultured without PBMCs. **(H)** Flow cytometry analysis of CD4^+^ T and CD8^+^ T cell proportions after co-culture. **(I)** ELISA quantification of TNF-α and IFN-γ levels in co-culture supernatants. ns, not significant; *P < 0.05; **P < 0.01; ***P < 0.001.

Next, we investigated the molecular mechanism by which ALKBH5 regulates PD-L1. Consistent with clinical observations, ALKBH5 knockdown markedly reduced PD-L1 protein expression (**Figures 5B, C, E, F**). Interestingly, PD-L1 mRNA levels were not significantly altered (**Figures 5A, D**), indicating that ALKBH5 primarily regulates PD-L1 at the post-transcriptional level, potentially by affecting mRNA stability or translation efficiency.

Finally, the immunomodulatory function of ALKBH5 was assessed using an *in vitro* co-culture system. Activated peripheral blood mononuclear cells were co-cultured with control (sh-NC) or ALKBH5 knockdown (sh-ALKBH5) MOLM-13 cells. Compared with sh-NC co-cultures, the proportion of CD8^+^ T cells was significantly increased in the sh-ALKBH5 co-cultures (*P* < 0.05, **Figure 5H**), and the secretion of T cell effector cytokines IFN-γ and TNF-α in the supernatant was also markedly elevated (*P* < 0.05, **Figure 5I**). Correspondingly, AML cells with ALKBH5 knockdown exhibited reduced survival in the co-culture system (*P* < 0.01, **Figure 5G**), indicating enhanced sensitivity to T cell-mediated cytotoxicity.

## 4 Discussion

The immunosuppressive bone marrow microenvironment is one of the major obstacles to the effective treatment of AML, and the aberrant expression of immune checkpoint molecules such as PD-L1 plays a key driving role in it (26–28). Although studies have been carried out on the transcriptional regulation of PD-L1, little is known about its post-transcriptional and epitranscriptomic regulatory mechanisms of expression in AML. The m6A demethylase ALKBH5 has been proven to be a key factor in immune regulation in solid tumors, yet its specific role and targets in AML remain unclear (20). This study fills this gap by revealing ALKBH5 as a novel post-transcriptional regulator of PD-L1 and its crucial role in immune evasion in AML.

In this study, bioinformatic analyses first revealed that ALKBH5 expression in AML patients is significantly higher than in normal hematopoietic stem cells, and that high ALKBH5 expression is associated with reduced overall survival, consistent with previous reports on ALKBH5 expression and prognosis in AML (25). Clinical sample validation further confirmed that ALKBH5 mRNA levels are markedly elevated in the bone marrow of AML patients compared to healthy controls. Notably, ALKBH5 expression was highest in the M1 subtype and lowest in the M4 subtype, indicating that a potential association may exist between ALKBH5 expression levels and disease heterogeneity across AML subtypes. Moreover, patients with high ALKBH5 expression exhibit a significantly higher proportion of peripheral blood blasts, with a trend toward increased bone marrow blasts. These findings suggest that ALKBH5 expression may be related to AML tumor burden and may play a role in malignant proliferation of AML cells. *In vitro* functional assays demonstrated that ALKBH5 knockdown in THP-1 and MOLM-13 AML cell lines significantly suppressed cell proliferation and migration, further supporting the role of ALKBH5 in promoting the malignant phenotype of AML cells. This aligns with the reported oncogenic functions of ALKBH5 in various solid tumors (29–31).

To explore the association between ALKBH5 and the AML immune microenvironment, GO and KEGG analyses revealed that differentially expressed genes between ALKBH5 high- and low-expression groups were significantly enriched in immune-related biological processes. These findings suggest that ALKBH5 may participate in the regulation of the AML immune microenvironment. Immune infiltration analysis showed that ALKBH5 expression was positively correlated with the infiltration levels of various immune cells such as CD8^+^ T cells, and positively correlated with the expression of immune checkpoint molecules such as PD-L1. Clinical sample analysis further validated the positive correlation between ALKBH5 and PD-L1 mRNA levels, with PD-L1 expression significantly higher in patients with high ALKBH5 expression compared to the low-expression group. Together, these results suggest that ALKBH5 may participate in AML immune regulation by modulating PD-L1 expression.

Subsequently, we constructed an ALKBH5-knockdown AML cell line and found a significant decrease in PD-L1 protein expression, while its mRNA level was not affected, suggesting that ALKBH5 regulates PD-L1 at the post transcriptional level. This regulatory pattern is consistent with findings by Zhang et al. in glioblastoma, which demonstrated that ALKBH5 enhances PD-L1 expression by reducing its degradation (32). A similar mechanism has also been reported in intrahepatic cholangiocarcinoma, where ALKBH5 was shown to increase PD-L1 mRNA stability by removing m6A modifications (20). Based on these findings, we speculate that in AML, ALKBH5 may maintain high PD-L1 expression by regulating the stability or translational efficiency of PD-L1 mRNA via its m6A demethylase activity.

In glioblastoma (32), ALKBH5 inhibitors enhance anti-tumor immune responses by downregulating PD-L1 protein expression and restore sensitivity in tumors that were initially resistant to PD-1 antibodies. In non-small cell lung cancer (33), ALKBH5 inhibition reduces PD-L1 expression in tumor cells and decreases M2 tumor-associated macrophage infiltration, thereby remodeling the immunosuppressive microenvironment and enhancing the efficacy of anti-PD-1 therapy. In melanoma (23), ALKBH5 deficiency decreases infiltration of regulatory T cells and myeloid-derived suppressor cells, enhances dendritic cell recruitment, and significantly inhibits tumor growth when combined with anti-PD-1 therapy; clinical data also indicate that patients with low ALKBH5 expression exhibit better responses to immunotherapy and improved survival outcomes. In this study, co-culture experiments of AML cells with activated PBMCs showed that ALKBH5 knockdown led to a significant increase in the proportion of CD8^+^ T cells, elevated secretion of IFN-γ and TNF-α in the supernatant, and a marked decrease in AML cell viability. These results suggest that ALKBH5 knockdown may enhance T cell-mediated anti-tumor immunity by downregulating PD-L1 expression on AML cells and relieving PD-1/PD-L1-mediated suppression of CD8^+^ T cell function, providing experimental evidence for the role of ALKBH5 in AML immune evasion.

Based on findings from studies in various tumors and our observations in AML that ALKBH5 knockdown relieves T cell suppression and enhances anti-tumor immunity, it is reasonable to hypothesize that combining ALKBH5 inhibition with anti-PD-1/PD-L1 therapy in AML may achieve synergistic effects through a dual blockade of immune evasion, by both reducing PD-L1 expression and alleviating T cell exhaustion. This hypothesis provides a novel potential strategy for combination therapy in AML, particularly for PD-L1-positive or immunotherapy-resistant cases, and warrants targeted experimental validation in future studies.

Furthermore, as an m6A demethylase, ALKBH5 has a broad range of target mRNAs, and its role in AML may not be limited to the regulation of PD-L1. Previous studies have shown that ALKBH5 can exert oncogenic effects in hepatocellular carcinoma and colorectal cancer by regulating target genes such as MAP3K8 and AXIN2 (22, 34), suggesting that ALKBH5 may coordinate multiple downstream pathways to modulate AML progression and the immune microenvironment. Future studies should employ integrated m6A-Seq and RNA-Seq analyses to systematically identify direct targets of ALKBH5 in AML and comprehensively elucidate its regulatory network.

Although this study systematically explored the role of ALKBH5 in AML, there are still several limitations. First, although we confirmed that ALKBH5 post-transcriptionally regulates PD-L1, the exact mechanism remains unclear, including whether it binds PD-L1 mRNA to remove m6A, affects mRNA stability or translation efficiency, or involves other factors like RNA-binding proteins. These issues need to be further validated through experiments such as RNA immunoprecipitation (RIP), m6A site directed mutagenesis and mRNA stability assays. Second, the sample size was limited and the distribution between AML patients and healthy donors was uneven, and subtype classification was mainly based on the FAB system. Future studies should include larger, better-balanced cohorts, apply updated classifications such as the WHO system, and conduct multicenter validation. Finally, functional experiments were primarily conducted *in vitro*, lacking *in vivo* animal model data, so the role of ALKBH5 in AML growth and immune evasion *in vivo* remains to be confirmed. Future studies could focus on developing humanized AML mouse models to assess the *in vivo* antitumor effects of ALKBH5, its role in regulating the immune microenvironment, and its therapeutic potential both as a monotherapy and in combination with PD-1/PD-L1 blockade.

In summary, this study provides preliminary evidence that ALKBH5 is highly expressed in AML and is associated with poor patient prognosis and elevated PD-L1 expression. ALKBH5 promotes AML cell proliferation and migration and may suppress CD8^+^ T cell function through post-transcriptional regulation of PD-L1, thereby contributing to immune evasion in AML. These findings offer new insights into the immunoregulatory mechanisms of AML; however, further experimental validation is required, and the feasibility of targeting ALKBH5 for AML immunotherapy warrants additional investigation.

## 5 Conclusion

In summary, ALKBH5 deficiency improves the immune microenvironment by increasing the number and proportion of CD8^+^ T lymphocytes. We further found that ALKBH5 knockdown activates the immune system, leading to enhanced production of antitumor cytokines IFN-γ and TNF-α. Moreover, ALKBH5 loss reduces PD-L1 protein levels *in vitro* without affecting its mRNA expression. Clinically, a positive correlation between ALKBH5 and PD-L1 was confirmed, highlighting the role of the ALKBH5-PD-L1 axis in AML immune evasion and providing new insights into the immunoregulatory mechanisms of AML. Given its oncogenic role in AML and its modulatory effects on the immune microenvironment, ALKBH5 holds potential as a therapeutic target for AML immunotherapy.

## Data Availability

The original contributions presented in the study are included in the article/**Supplementary Material**. Further inquiries can be directed to the corresponding author.
