# Supplementary material for: ALKBH5 facilitates acute myeloid leukemia development and immune escape via PD-L1 regulation
Source: Front Oncol. 2026 Feb 26;16:1781803. doi: 10.3389/fonc.2026.1781803 (PMC12979091; doi:10.3389/fonc.2026.1781803)
Supplement: Supplementary file 1 [file DataSheet1.docx]

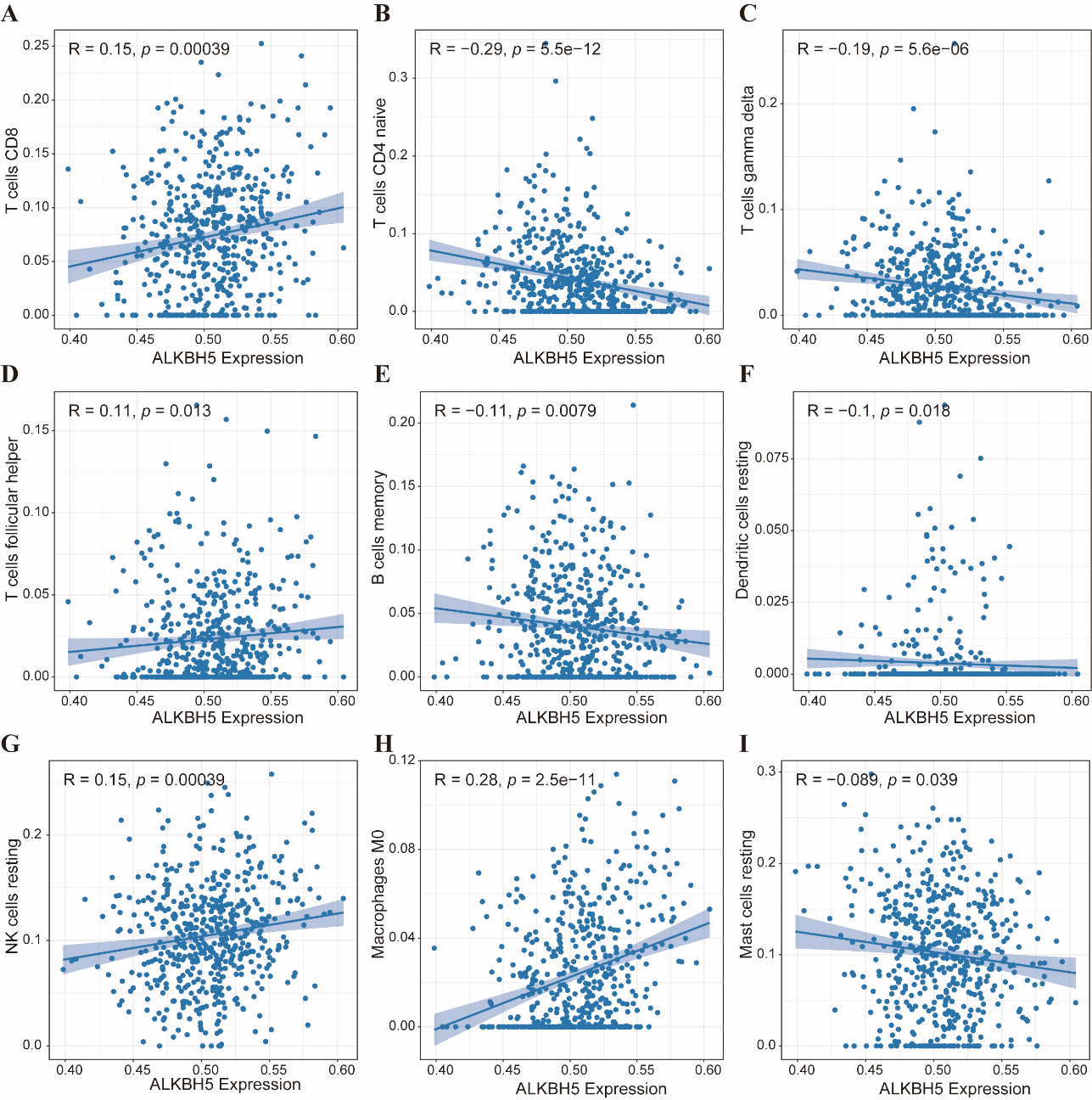


**Figure S1**. Pearson correlation analyses between ALKBH5 expression and immune cell infiltration in AML.

(A) Correlation between ALKBH5 expression and CD8⁺ T cells.

(B) Correlation between ALKBH5 expression and naïve CD4⁺ T cells.

(C) Correlation between ALKBH5 expression and γδ T cells.

(D) Correlation between ALKBH5 expression and T follicular helper cells.

(E) Correlation between ALKBH5 expression and memory B cells.

(F) Correlation between ALKBH5 expression and resting dendritic cells.

(G) Correlation between ALKBH5 expression and resting NK cells.

(H) Correlation between ALKBH5 expression and M0 macrophages.

(I) Correlation between ALKBH5 expression and resting mast cells.


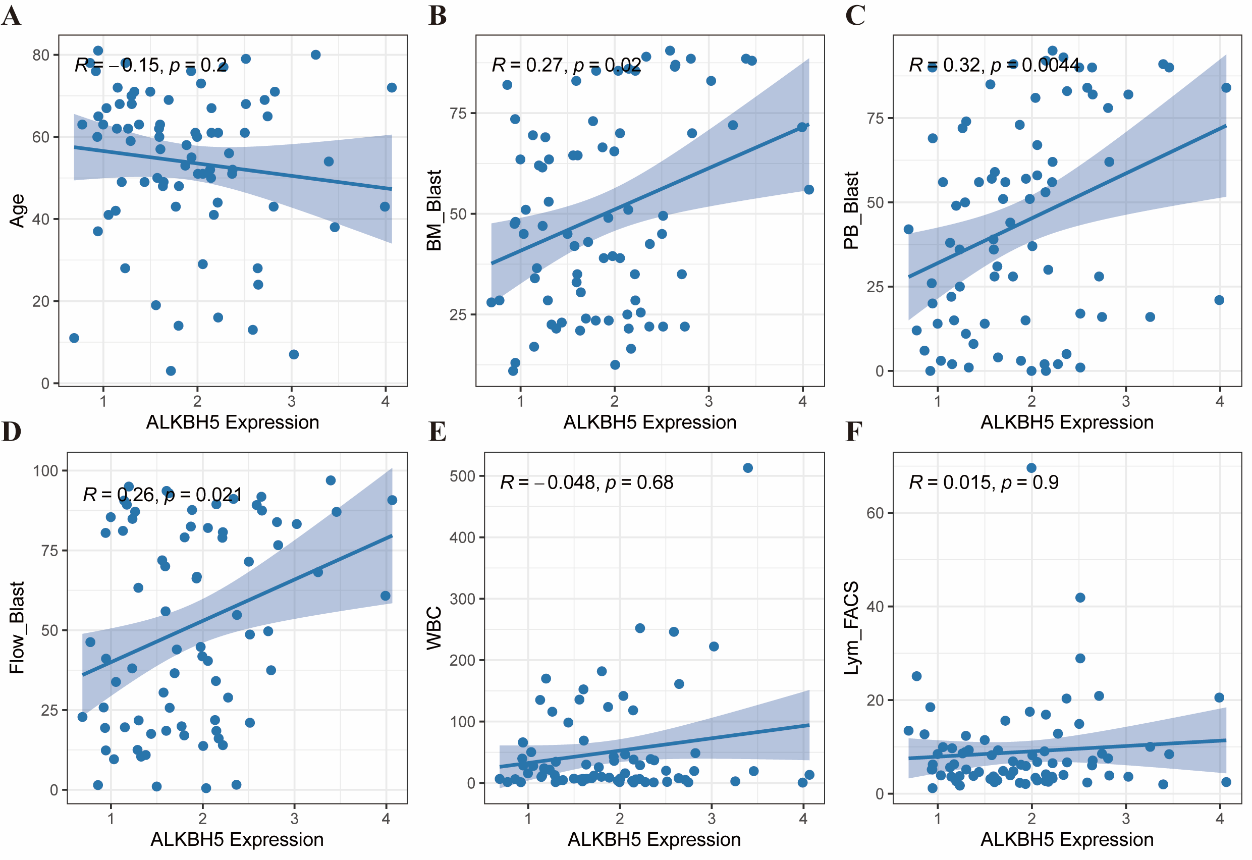


**Figure S2**. Spearman correlation analyses between ALKBH5 expression and clinical characteristics in AML patients.

(A)Correlation between ALKBH5 expression and patient age.

(B) Correlation between ALKBH5 expression and the percentage of bone marrow blasts (BM_Blast).

(C) Correlation between ALKBH5 expression and the percentage of peripheral blood blasts (PB_Blast).

(D) Correlation between ALKBH5 expression and the percentage of bone marrow blasts detected by flow cytometry (Flow_Blast).

(E) Correlation between ALKBH5 expression and white blood cell count (WBC).

(F) Correlation between ALKBH5 expression and the percentage of lymphocytes in bone marrow measured by flow cytometry (Lym_FACS).
