# Supplementary material for: ALKBH5 facilitates acute myeloid leukemia development and immune escape via PD-L1 regulation
Source: Front Oncol. 2026 Feb 26;16:1781803. doi: 10.3389/fonc.2026.1781803 (PMC12979091; doi:10.3389/fonc.2026.1781803)
Supplement: Supplementary file 2 [file Table1.docx]

**Table S1. Clinical characteristics of the study cohorts**

| **Characteristic** | **Total**  **(N = 90)** | **AML**  **(N = 77)** | **Control**  **(N = 13)** | ***p*** |
| --- | --- | --- | --- | --- |
| Age, median (Q1–Q3) | 59.50(45.00; 67.00) | 59.00(44.00; 68.00) | 60.00(55.00; 65.00) | 0.922 |
| Sex, n (%) |  |  |  | 1.000 |
| Female | 40 (44.44%) | 34 (44.16%) | 6 (46.15%) |  |
| Male | 50 (55.56%) | 43 (55.84%) | 7 (53.85%) |  |

**Table S2.** **shRNA sequences used in this study**

| **sh-RNA** | **Sequences** (5‘ – 3’) |
| --- | --- |
| sh-ALKBH5#1 | GGATCCTGACGTCCCGGGACAACTATACTCGAGTATAGTTGTCCCGGGACGTCATTTTTTGAATTC |
| sh-ALKBH5#2 | GGATCCTCGTGTCCGTGTCCTTCTTTACTCGAGTAAAGAAGGACACGGACACGATTTTTTGAATTC |
| sh-ALKBH5#3 | GGATCCTGGATATGCTGCTGATGAAATCTCGAGATTTCATCAGCAGCATATCCATTTTTTGAATTC |

**Table S3. Primer sequences used for real-time quantitative PCR in this study**

| **Gene** | **Sequence (F: 5′-3′)** | **Sequence (R: 5′-3′)** |
| --- | --- | --- |
| ALKBH5 | CGAGGACCTGGTGTTTCTA | GGTTCTCTTCTTGTTCATCTC |
| PD-L1 | CCAGTCACCTCTGAACATGAA | ATTGGTGGTGGTGGTCTTAC |
| β-actin | CACTCTTCCAGCCTTCCTTC | GTACAGGTCTTTGCGGATGT |
